# Divergent Expression of Acidic and Alkaline Pectate Lyases in *Ditylenchus destructor* During Initial Infection Time Course

**DOI:** 10.3390/microorganisms14040829

**Published:** 2026-04-04

**Authors:** Bingxue Sun, Bo Gao, Yonghao Dong, Xiuhua Li, Juan Ma, Rongyan Wang, Shulong Chen

**Affiliations:** National Collection of Plant-Associated Microbes (Hebei), IPM Innovation Center of Hebei Province, International Science and Technology Joint Research Center on IPM of Hebei Province, Plant Protection Institute, Hebei Academy of Agriculture and Forestry Sciences, Baoding 071000, China; sunbingxuechina@163.com (B.S.); gaobo89@163.com (B.G.); dongyonghao1119@163.com (Y.D.); lixiuhua727@163.com (X.L.); chenshulong65@163.com (S.C.)

**Keywords:** *Ditylenchus destructor*, pectate lyase, enzymatic activity, pH

## Abstract

Pectate lyase (PeL) is a key cell wall-degrading enzyme in the infection process of plant-parasitic nematodes, with a large gene family exhibiting functional redundancy. The dominant PeL isoform during the initial infection time course remains unclear. In this study, 21 *Ddpel* genes were identified in *Ditylenchus destructor* Thorne, 1945, 7 of which were differentially expressed during the initial infection time course of this nematode. The purified proteins of these seven DdPeLs showed pathogenicity toward both sweet potato and tobacco, and their optimal enzymatic pH varied significantly. Prior to host infection, *D. destructor* preferentially expresses *Ddpel* genes encoding pectate lyase with higher activity at pH 5.8. However, within 5 days post-inoculation with nematodes, the expression of genes encoding acidic DdPeL enzymes (enzymes with optimal activity in acidic pH) was upregulated, while genes encoding alkaline DdPeL enzymes (optimal activity in alkaline pH) were concurrently downregulated. Through site-directed mutagenesis, we demonstrated that the loss of enzymatic activity in DdPeLs abolished their ability to induce plant cell death. Furthermore, when acidic or alkaline DdPeLs were pre-treated with dialysis in their respective optimal pH buffers prior to infiltration, their pathogenicity was significantly enhanced. Together, these findings demonstrate that enzymatic activity, governed by protein structure and local pH, is a key determinant of pathogenicity. Previous studies have reported that phytopathogens can secrete organic acids during the initial infection phase, leading to localized acidification of the host microenvironment. We therefore hypothesize that, during the initial infection time course, nematodes may actively acidify the host microenvironment to specifically enhance the enzymatic activity of acidic DdPeLs, thereby promoting cell wall degradation and facilitating infection establishment.

## 1. Introduction

The potato rot nematode, *Ditylenchus destructor* Thorne, 1945, ranks among the most devastating pathogens of sweet potato (*Ipomoea batatas* (L). Lam, 1793) and is the second most significant plant-parasitic nematode found in potato (*Solanum tuberosum* L. 1753) [1]. This nematode infects over 30 crop species across 10 families, including sweet potato, potato, Chinese angelica (*Angelica sinensis* (Oliv.) Diels, 1900), and Codonopsis (*Codonopsis pilosula* (Franch.) Nannf., 1929), causing escalating economic losses worldwide [2]. Yield losses attributed to *D. destructor* typically range from 20% to 50% and can reach 100% in endemic regions. In China, the frequent transportation of sweet potato seed tubers and seedlings facilitates disease spread, often resulting in severe yield reduction or even total crop loss, posing a major threat to the sustainable development of the sweet potato industry [2]. Due to its severe impact and persistence once established in fields, *D. destructor* has been designated as a significant quarantine pest in numerous countries (European and Mediterranean Plant Protection Organization, EPPO, www.eppo.org, accessed on 9 June 2024). Despite its status as a destructive plant-parasitic nematode threatening global food security [3], effective control measures for *D. destructor* remain limited. Consequently, elucidating its pathogenic mechanisms is imperative for developing novel control strategies.

Successful host invasion by nematodes is fundamental to pathogenicity, and breaching the plant cell wall barrier represents one of the most critical steps during early infection [4]. Cell wall-degrading enzymes (CWDEs), acting as “molecular scissors,” are primarily responsible for degrading the polysaccharide network of plant cell walls [4,5,6]. Unlike sedentary endoparasitic nematodes (e.g., *Meloidogyne incognita* (Kofoid and White, 1919) Chitwood, 1949), migratory endoparasitic nematodes, such as *D. destructor*, lack fixed feeding sites due to their requirement for dynamic penetration through host tissues. Consequently, they exhibit a greater reliance on the continuous secretion of CWDEs, such as pectate lyase [7]. Comparative genomic studies reveal significant expansions in the CWDE gene family within migratory nematodes [8]. For instance, both the copy number and expression levels of pectate lyase and cellulase genes are typically higher in migratory species than in their sedentary counterparts, aligning with their distinct infection strategies [9,10].

pH serves as a critical environmental factor governing the catalytic function of pathogen-derived CWDEs [11,12]. Studies have demonstrated that most pathogen-secreted CWDEs exhibit optimal activity under weakly acidic conditions [13]. For instance, pectin, cellulose-, and hemicellulose-degrading enzymes display the highest catalytic efficiency in a weakly acidic apoplast, enabling the rapid hydrolysis of polysaccharide components in the plant cell wall [14,15]. To maximize pathogenicity, diverse pathogens have evolved strategies to actively remodel apoplastic pH: *Botrytis cinerea* Pers., 1801, and *Sclerotinia sclerotiorum* (Lib.) de Bary, 1884, secrete organic acids, including oxalic acid and citric acid, to directly reduce apoplastic pH [16,17], while *Fusarium oxysporum* Schltdl., 1824, releases fusaric acid for the same purpose [16]. Some pathogens can also hijack host plasma membrane H^+^-ATPases to induce apoplastic acidification, thereby creating an optimal catalytic milieu for CWDEs [18,19]. Plants rapidly trigger apoplastic alkalinization upon the perception of pathogen-associated molecular signals [20,21]. Recent studies have revealed that apoplastic alkalinization directly suppresses the catalytic activity of pathogen CWDEs by altering their protein conformations and the microenvironment of their catalytic sites, thus delaying cell wall degradation. In *Arabidopsis thaliana* (L.) Heynh., 1842, *CPK3/9/29/33* phosphorylate the Ser899 residue of the plasma membrane H^+^-ATPases *AHA1/2* to induce immune-triggered apoplastic alkalinization, a signal that propagates to distal uninfected tissues [21]. Current research in this field has predominantly focused on bacterial and fungal pathogens. However, in plant-parasitic nematodes, it remains unclear whether nematodes secrete acidic CWDEs to facilitate infection during the initial infection time course.

Pectate lyase (PeL; EC 4.2.2.2), a key CWDE, specifically cleaves the α-1,4-glycosidic bonds of pectic polysaccharides via a β-elimination reaction. This action disrupts the middle lamella and tissue integrity, making PeL crucial for nematode penetration and migration [5]. PeL has been established as an essential pathogenicity factor in various plant-parasitic nematodes [2,5,20,22]. RNA interference experiments robustly demonstrate the virulence role of PeL: silencing the *Bxpel1* gene in the pine wood nematode (*Bursaphelenchus xylophilus* (Steiner and Buhrer, 1934) Nickle, 1970) significantly reduced its migration speed, reproductive capacity, and pathogenicity by 71.6%, 98.3%, and 44.4%, respectively [23]; similarly, silencing the *Hg-pel-6* gene in the soybean cyst nematode (*Heterodera glycines* Ichinohe, 1952) decreased the number of invading nematodes by 46.9% and suppressed female development by 61.5% [24]. PeLs typically exist as multigene families with functional redundancy among members [25,26]. For instance, four *pel* genes were characterized in the burrowing nematode *Radopholus similis* (Cobb, 1893) Thorne, 1949 [27], and seven *pel* genes were found in *H. glycines* [24]. Studies also indicate that genes encoding PeLs with pectin-degrading and hydrolytic activities are undergoing rapid expansion in migratory endoparasitic nematodes [8,28]. Current research on pectate lyase has predominantly focused on individual PeL functions [29]. For example, VdPeL1 triggers defense responses, inducing resistance in tobacco and cotton plants against *Botrytis cinerea* and *V. dahlia* [18]. The bean protein PvPGIP2 can hijack the fungal pectinase FpPG to reshape oligogalacturonide product profiles [30]. However, which specific PeL isoforms are selectively secreted by the nematode during the initial infection time course is unknown.

This study investigates the expression patterns of pectate lyase genes during the initial infection time course of *D. destructor*. Employing genome-wide identification, transient expression assays in tobacco, prokaryotic expression, enzymatic activity measurements, and transcriptome analysis, we report three key observations: (1) the DdPeL enzymatic activity level is the primary determinant of the phenotypic outcome in tobacco; (2) prior to host incultion, *D. destructor* preferentially expresses *Ddpel* genes encoding pectate lyase with higher activity at pH 5.8; and (3) the genes encoding acidic DdPeL enzymes was upregulated during the initial infections time course (0–5 days), downregulating those encoding alkaline DdPeL enzymes. Previous studies have reported that phytopathogens can secrete organic acids during the initial infection phase, leading to localized acidification of the host microenvironment., We therefore hypothesize that, during the initial infection time course, nematodes may actively acidify the host microenvironment to specifically enhance the enzymatic activity of acidic DdPeLs, thereby promoting cell wall degradation and facilitating infection establishment.

## 2. Materials and Methods

### 2.1. Maintenance and Propagation of D. destructor

*Ditylenchus destructor* isolates were maintained in our laboratory. The nematodes were preserved and propagated in vivo using the carrot disk culture method, as described previously [31]. Two distinct *D. destructor* populations, designated A and B, were utilized in this study. Population A, isolated from sweet potato, served as the principal experimental subject. Population B, isolated from potato, was employed to additionally corroborate the findings related to the upregulation of genes encoding acidic pectate lyases during the initial stages of infection.

### 2.2. Identification of the Pectate Lyase Gene Family in D. destructor A Population

The pectate lyase gene family was identified through the following procedure: Initially, all candidate genes annotated as pectate lyases were extracted from the genome annotation file (https://parasite.wormbase.org/index.html BioProject ID: PRJNA800207, accessed on 12 June 2024). Subsequently, the conserved domains of the corresponding protein sequences were verified using the Pfam (https://www.ebi.ac.uk/interpro/, accessed on 14 June 2024) or NCBI-CDD database (https://www.ncbi.nlm.nih.gov/cdd, accessed on 15 June 2024) to confirm the presence of characteristic pectate lyase domains. The parameters were set to default. The validated genes were further analyzed as follows: TBtools (version 2.390) was employed to align their CDSs with the corresponding genomic sequences using the GXF Re-build from Sequences function, with the Search Range set to 20,000 and Max Pre Hits set to 1000, to generate gene structure diagrams illustrating the composition and distribution of introns and exons. In parallel, the MEME suite (version 5.5.3) was used for the motif analysis of these protein sequences under default parameters, aiming to reveal conserved motif features within the gene family.

### 2.3. Heterologous Expression of Pectate Lyase

The pectate lyase was expressed and purified using a prokaryotic expression system. The gene encoding the target enzyme was cloned into the pET-28a(+) expression vector, and the recombinant plasmid was transformed into *Escherichia coli* BL21(DE3) competent cells. A single colony was inoculated into LB medium containing kanamycin and cultured at 37 °C with shaking until the OD_600_ reached approximately 0.6. Protein expression was induced with 0.5 mM IPTG at 16 °C for 20 h. The cells were harvested, disrupted by sonication, and centrifuged to collect the supernatant. The His-tagged recombinant protein was purified using nickel-affinity chromatography, eluted with a buffer containing different concentrations of imidazole (100 mM–500 mM), and subsequently dialyzed into a PBS buffer. Protein concentration was determined using a BCA protein assay kit (Coolaber, Beijing, China), with bovine serum albumin as the standard. The absorbance at 562 nm was measured using a microplate reader (Biotek, Synergy H1, Winooski, VT, USA), and the concentration was calculated from a standard curve.

### 2.4. RNA-Seq Transcriptome Analysis

RNA-seq data were obtained from *D. destructor* A and B populations sampled at different time points following inoculation on sweet potato cv. Long9 (a susceptible cultivar maintained in the laboratory) callus, including non-inoculated nematodes (0 d), as well as nematodes at 1, 3 and 5 days post-inoculation. After inoculation, the tubers were placed at room temperature. At each time point, inoculated tubers were thoroughly rinsed three times with sterile deionized water to remove nematodes feeding on the surface. The inoculation sites were then placed upside down in sterile water for 5–10 min to collect nematodes that had not deeply invaded, based on their hydrotaxis. The collected nematodes were centrifuged at 12,000 rpm, and the pellet was immediately frozen in liquid nitrogen and stored at −80 °C until RNA extraction. Each time point contained three biological replicates, with approximately 80,000 nematodes pooled per replicate.

### 2.5. Relative Expression of Ddpel Genes

To verify the expression trends observed in the RNA-seq results, quantitative real-time PCR (qPCR) was performed. Approximately 20,000 *D. destructor* were inoculated onto a sweet potato callus. Nematodes were collected at 0, 1, 3 and 5 days post-inoculation. The method for nematode collection was the same as described above. Total RNA was extracted from the collected nematodes and reverse-transcribed into cDNA. The resulting cDNA was used as a template for qPCR. The expression trends observed in the RNA-seq results were further verified by quantitative real-time PCR (qPCR) using the 2^−ΔΔCT^ method for accurate relative quantification. For data normalization, *EF-1* was used as an internal reference gene. qPCR was performed with three biological replicates and three technical replicates per sample. The primer sequences used for qPCR are listed in Table S4.

### 2.6. Pathogenicity Assay of Purified Pectate Lyase

To assess the pathogenicity of the purified pectate lyase, infection assays were carried out using a sweet potato cv. Long9 callus and *Nicotiana benthamiana* Domin, 1929, leaves. The purified enzyme solution (0.3–0.5 mg/mL) was drop-inoculated onto the surface of sweet potato callus, with an equal volume of buffer serving as the control. The inoculated callus was incubated at 25 °C under 70–80% humidity for 2–5 days, after which lesions were photographed. For tobacco assays, the same enzyme solution was infiltrated into *N. benthamiana* leaves using a sterile syringe, while control leaves were infiltrated with the buffer. To further examine the effect of different buffer conditions on enzymatic activity, the pectate lyase was dialyzed in buffers at various pH levels (Solarbio Biotech, Beijing, China). The dialyzed enzyme solutions were then infiltrated into tobacco leaves, with the corresponding buffers used as controls. Lesion development was observed and photographed (SonyA5000, Tokyo, Japan) 2–5 days post-infiltration. Each experiment was performed in triplicate.

### 2.7. Agrobacterium-Mediated Transient Expression

Different fragments of *Ddpels* were amplified using wild-type *D. destructor* A population’s cDNA as the template. The fragments were inserted into ClaI/NotI-digested PVX using the infusion method and verified by sequencing (Sangon Biotech, Shanghai, China). The recombinant plasmids were transformed into *Agrobacterium tumefaciens* strain GV3101 (pJIC SA_Rep). For agroinfiltration assays, leaves from 4- to 5-week-old *N. benthamiana* plants grown at 23 °C were used. The recombinant empty vector (PVX-GFP) was used as a negative control. *Nicotiana benthamiana* was observed for 7 days and photographed on the seventh day after inoculation. Ten *N. benthamiana* were infiltrated per recombinant plasmid. Type I (typical mosaic symptoms with dark light-green patches) and Type II symptoms (vascular browning) were examined and photographed using a stereomicroscope (Leica M165 C, Wetzlar, Germany). The fresh tissue sections without staining were observed.

### 2.8. Assay of DdPeL Activity

Pectate lyase activity was determined by measuring the increase in absorbance at 235 nm, corresponding to the formation of unsaturated bonds. For each tested pH condition, the reaction mixture was prepared separately. It consisted of 190 µL of a specific buffer containing 0.2% polygalacturonic acid (PGA) and 0.1 mM CaCl_2_, to which 10 µL of diluted enzyme solution (0.1–1 mg/mL) was added. The assays were performed individually across the following buffer systems: 50 mM sodium acetate-acetic acid (NaAc-HAc) at pH 3.8, 4.5, and 5.8; 50 mM Tris-HCl at pH 6.8 and 7.4; and 50 mM glycine-NaOH at pH 8.8, 9.0, and 10.0. The reaction was carried out at 45 °C for 30 min and terminated immediately by adding 300 µL of 0.03 M phosphoric acid (H_3_PO_4_). Three replicates were performed for each treatment.

### 2.9. Electrolyte Leakage Rate

The electrolyte leakage rate was determined using *N. benthamiana*. Fresh samples (stems, petioles, or leaves) were cut into uniform pieces of approximately 5 mm. Equal masses of tissues were combined for each replicate sample. The tissue samples were thoroughly rinsed three times with deionized water to remove surface electrolytes. Subsequently, they were immersed in a beaker containing 30 mL of deionized water. The beaker was incubated at room temperature (25 °C) on a shaker at 100 rpm for 4 h. The initial electrical conductivity (EC1) of the bathing solution was measured using a conductivity meter (HI9932, Hanna Instruments, Smithfield, RI, USA). The samples were heated to 100 °C for 20 min to release all intracellular electrolytes. After cooling to room temperature, the final electrical conductivity (EC2) was measured. Three replicates were performed for each treatment. The electrolyte leakage rate was calculated as a percentage using the formula: electrolyte leakage (%) = (EC1/EC2) × 100.

### 2.10. Determination of pH in Healthy and Diseased Plant Tissues

Sweet potato seedlings (resistant cultivars: su24 and 21156; susceptible cultivar: Long9) were planted in an experimental field containing *D. destructor* population A. After tuber formation (after approximately 4 months), healthy and diseased tubers were collected. For healthy tissue samples, visually healthy tubers were selected, and the intact tuber tissue was excised. For diseased tissue samples, the symptomatic lesion areas were excised from infected tubers. To confirm that the observed symptoms were specifically caused by *D. destructor*, symptomatic tubers were examined for typical “spongy, whitish internal rot” symptoms (Figure S4), followed by microscopic observation to verify the presence of *D. destructor*. Genomic DNA was extracted from diseased tissues. The ITS region was amplified using primers ITS1/ITS4 (for fungi and plants), TW81/AB28 (for nematodes), and the 16S rRNA gene using primers 27F/1492R (for bacteria) (Table S4). All amplicons were sequenced (Sangon Biotech Co., Ltd., Shanghai, China) to confirm that the symptoms were mainly caused by *D. destructor*. All amplicons were cloned and sequenced (Sangon Biotech Co., Ltd., Shanghai, China) to confirm that the symptoms were caused by *D. destructor*. The pH of healthy and diseased plant tissues was measured as follows. Equal amounts of the respective tissues were accurately weighed and homogenized in a pre-chilled sterile mortar with ice-cold sterile deionized water in a 1:1 (*w*/*v*) ratio. The homogenate was filtered through four layers of sterile gauze, and the clarified extract was retained. Using a precision pH meter (FiveEasy Plus, METTLER TOLEDO, Greifensee, Switzerland) calibrated with standard buffers, the electrode was immersed in the center of the supernatant. The pH value was recorded after the reading stabilized, with at least three biological replicates per sample group.

### 2.11. Statistical Analysis

All experiments were performed with three independent biological replicates, each consisting of three technical replicates. Data are presented as mean ± standard deviation. Statistical analyses were conducted using SPSS software (version 23.0, IBM Corp., Armonk, NY, USA). Pearson’s correlation coefficient was used to evaluate correlations between variables. For comparisons among multiple groups, one-way analysis of variance (ANOVA) was performed, followed by Fisher’s Least Significant Difference test for multiple comparisons.

## 3. Results

### 3.1. Identification of Pectate Lyase Gene Family

**Figure 1:**
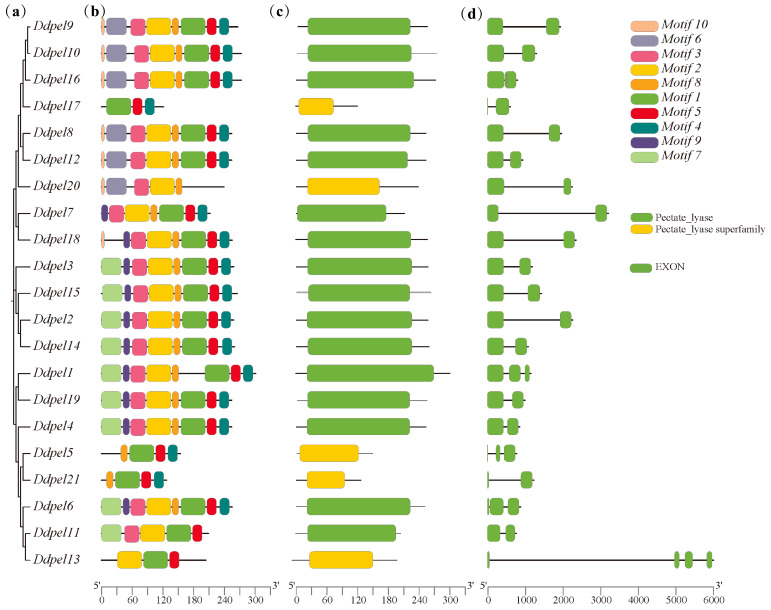
Identification of the pectate lyase gene family. (**a**) the phylogenetic tree constructed from protein sequences; (**b**) the distribution of conserved motifs identified using the MEME suite, with each motif represented by a numbered colored box. Black horizontal lines represent the amino acid sequence length of individual proteins, and the ruler at the bottom is the scale bar for amino acid length.; (**c**) the positions of pectate lyase domains within the full-length protein sequences. Black horizontal lines represent the amino acid sequence length of individual proteins, and the ruler at the bottom is the scale bar for amino acid length; and (**d**) the exon-intron organization of the corresponding genes, where exons are shown as rectangles and introns as lines. Black horizontal lines represent the nucleotide sequence length of individual *DdPel* genes, and the ruler at the bottom is the scale bar for nucleotide length.

To identify pectate lyase genes within the *D. destructor* genome, the protein sequence of the pectate lyase domain served as the query in a BLAST (https://www.ncbi.nlm.nih.gov/cdd, accessed on 15 June 2024) search. This analysis identified 21 pectate lyase genes (Figure 1). These genes comprise 2–4 exons and encode proteins ranging from 121 to 273 amino acids. Among these, 16 members are predicted to possess an N-terminal signal peptide (Table S1), suggesting their potential for secretion and likely apoplastic localization. The theoretical isoelectric points (pI) of these proteins span a broad range (4.0–8.28) (Table S1). This observed pI diversity, coupled with the inherent redundancy of pectate lyases, may facilitate the secretion of specific enzyme isoforms tailored to distinct host and environmental conditions, thus enabling the efficient degradation of the host plant cell wall.

### 3.2. Recombinant Pectate Lyase Is Pathogenic to Sweet Potato and Tobacco

**Figure 2:**
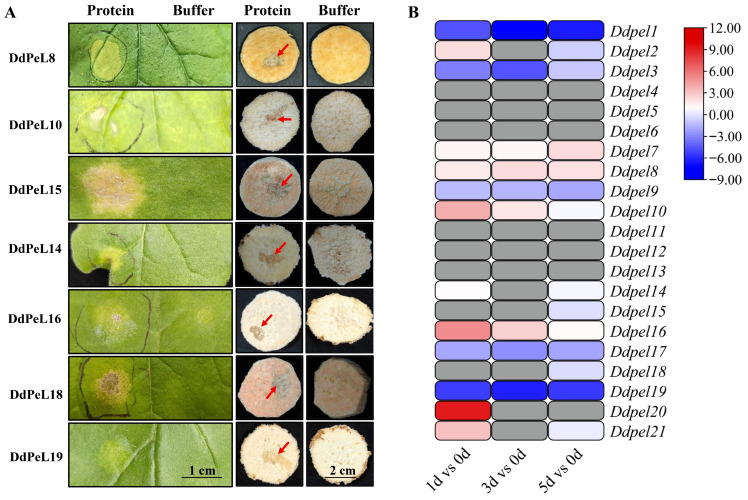
The recombinant pectate lyase is pathogenic to sweet potato and tobacco. (**A**) The functional validation of recombinant pectate lyase protein. The purified prokaryotically expressed protein was dialyzed in a buffer at the optimal pH for enzymatic activity, then injected/infiltrated into tobacco leaves and inoculated onto sweet potato callus tissues. Photographs were taken 3–7 days post-inoculation. The red arrow indicates the degradation of the sweet potato cell wall by the enzyme, while the yellowish lesions observed in tobacco leaves similarly represent cell wall degradation caused by the enzyme. (**B**) The expression profiles of 21 pectate lyase genes in sweet potato during 0–5 days of inoculation with *Ditylenchus destructor*. The heatmap displays log_2_(fold change) values of gene expression compared to the non-inoculated control (0 day). Gene expression levels are represented in a heatmap, with red indicating upregulation, blue indicating downregulation, and gray denoting no expression. Darker shades indicate greater changes in expression.

Transcriptome analysis was performed to identify the differential expression of *Ddpel* genes during the initial infection time course (1d, 3d, and 5d) of *D. destructor*. Based on the criteria |log_2_(fold change)| > 1 and the presence of an N-terminal signal peptide, a total of 12 differentially expressed genes were identified: *Ddpel1*, *Ddpel2*, *Ddpel3*, *Ddpel8*, *Ddpel9*, *Ddpel10*, *Ddpel14*, *Ddpel15*, *Ddpel16*, *Ddpel18*, *Ddpel19*, and *Ddpel20* (Figure 2B; Table S1). These 12 genes were selected for prokaryotic expression in *E. coli*. Of these *Ddpel* genes, seven proteins were successfully expressed and purified (Figure S1; up-regulation: *Ddpel8*, *Ddpel10*, *Ddpel14* and *Ddpel16*; down-regulation: *Ddpel15*, *Ddpel19* and *Ddpel18*). The remaining five genes, including *Ddpel1*, *Ddpel2*, *Ddpel3, Ddpel9*, and *Ddpel20*, could not be successfully expressed and were therefore excluded from further analysis. Pathogenicity assays via *N. benthamiana* leaf infiltration and detached *I. batatas* tuber inoculation confirmed in vitro pathogenic activity for all seven enzymes, with each inducing necrotic lesions (Figure 2A).

### 3.3. Enzymatic Activities and Optimal pH of DdPeLs

**Figure 3:**
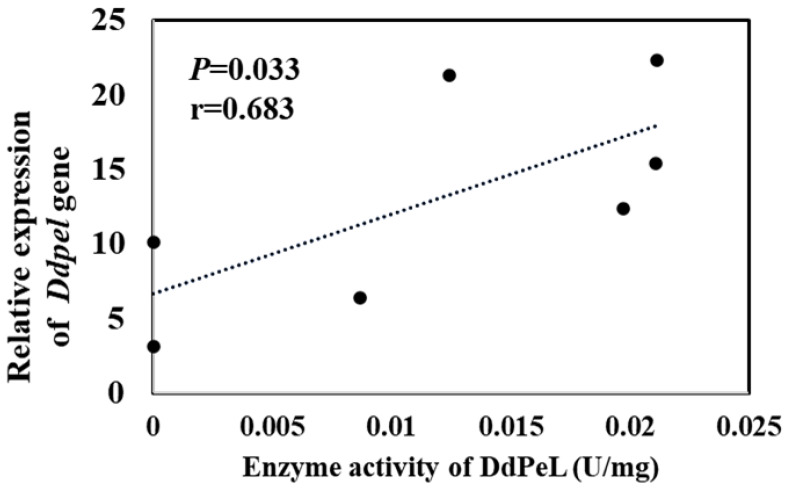
The correlation analysis between the expression levels (non-inoculated) of pectate lyase genes and pectate lyase activities at pH 5.8. Pearson’s correlation coefficient was used to evaluate correlations between variables. A *p* value of less than 0.05 indicates a significant correlation, with r representing the strength of the correlation.

To determine whether the different DdPeLs exhibit pectate lyase activity, each gene was heterologously expressed in *E. coli* BL21(DE3), and the corresponding protein was purified (Figure S1). Enzymatic assays showed that all seven DdPeLs were capable of degrading polygalacturonic acid, with activities ranging from 0.019 to 0.170 U mg^−1^ (Table S2). Further analysis of their activities under different pH conditions revealed distinct optimal pH values for each enzyme: DdPeL15, DdPeL16, DdPeL18, and DdPeL19 displayed optimal activity under alkaline conditions (pH 9.0, 9.0, 9.0, and 8.8, respectively), whereas DdPeL8, DdPeL10, and DdPeL14 showed optimal activity under acidic to neutral conditions (pH 3.8, 5.8, and 7.4, respectively; Table 1). These results demonstrate functional divergence within the DdPeL family in terms of both catalytic activity and pH adaptation. The pH of healthy host tissues ranged from 5.8 to 6.3 (Figure S3). When assayed at pH 5.8, the enzymatic activities of DdPeLs showed a significant positive correlation with *Ddpel* gene expression levels during the pre-infection stage (*p* < 0.05; Figure 3), whereas no significant correlations were observed at other pH values (Figure S2).

### 3.4. Expression of Acidic Pectate Lyase Gene Was Induced During Initial Infection Stage

**Figure 4:**
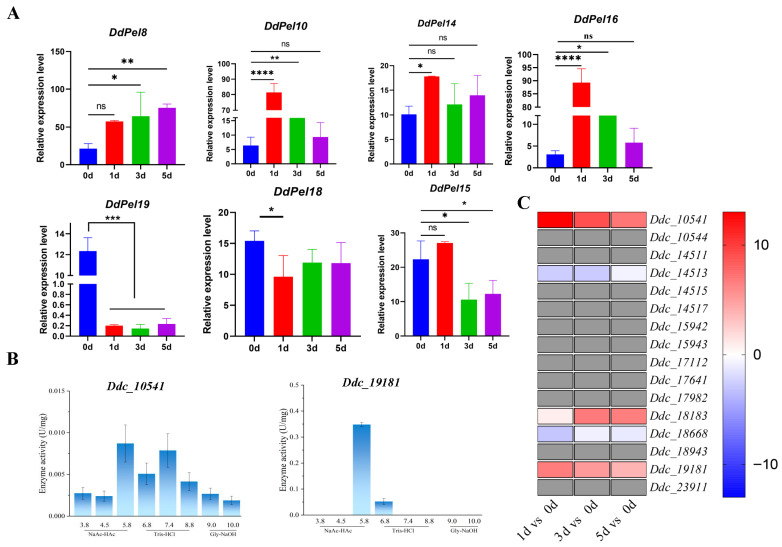
The expression of the acidic pectate lyase gene was induced during the initial infection stage. (**A**) The expression levels of acidic/alkaline pectate lyase genes (determined by qPCR). For data normalization, *EF-1* was used as an internal reference gene. For comparisons among multiple groups, one-way analysis of variance was performed, followed by Fisher’s Least Significant Difference test for multiple comparisons. Data are presented as mean ± standard deviation. Statistical significance is indicated as follows: * *p* < 0.05, ** *p* < 0.01, *** *p* < 0.001, and **** *p* < 0.0001. ns indicates not significant. (**B**) The enzymatic activities of two pectate lyases (*Ddc_10541* and *Ddc_19181*) upregulated in *D. destructor* population B, measured across a range of pH values. Data are presented as mean ± standard deviation. (**C**) The expression profiles of pectate lyase genes in *D. destructor* population B (derived from RNA-seq data). The heatmap displays log_2_(fold change) values of gene expression compared to the non-inoculated control (0 day). The color scale ranges from blue (downregulation) through white (no change) to red (upregulation). Darker shades indicate greater changes in expression. Gray indicates no detectable expression.

As pectate lyases serve as critical virulence determinants during the initial infection’s time course, characterizing their initial expression patterns was essential [5,6,7]. Time-course analysis of pectate lyase gene expression during inoculation (1, 3, 5) demonstrated the following: (1) acidic pectate lyase genes (*Ddpel8*, *Ddpel10*, and *Ddpel14*) were significantly upregulated; and (2) alkaline pectate lyase genes (*Ddpel19*, *Ddpel18*, and *Ddpel15*) were progressively downregulated (Figure 4A). The populations of *D. destructor* are known to vary in pathogenicity [2], and genomic comparisons have revealed differences in the number and sequence similarity of cell wall-degrading enzymes across populations [32]. To test whether the upregulation of acidic pectate lyases occurs in other *D. destructor* population, the transcriptomic data and genome annotation (https://www.ncbi.nlm.nih.gov/datasets/genome/GCA_022814895.1/, accessed on 12 June 2024) from *D. destructor* B population were used. Based on the criterion that log_2_(fold change) > 1 at each of the three time points (1, 3, and 5 days post-inoculation) compared to the non-inoculated control (0 d), two pectate lyase genes (*Ddc_10541* and *Ddc_19181*) were identified as highly upregulated during the initial infection time course (Figure 4C). These two genes were selected for functional characterization. Enzymatic profiling revealed optimal activity under acidic conditions (pH 5.8) for both isoforms (Figure 4B), consistent with the observations in population A.

### 3.5. Alkaline Pectate Lyase Induces More Severe Necrosis in N. benthamiana than Acidic Pectate Lyase

**Figure 5:**
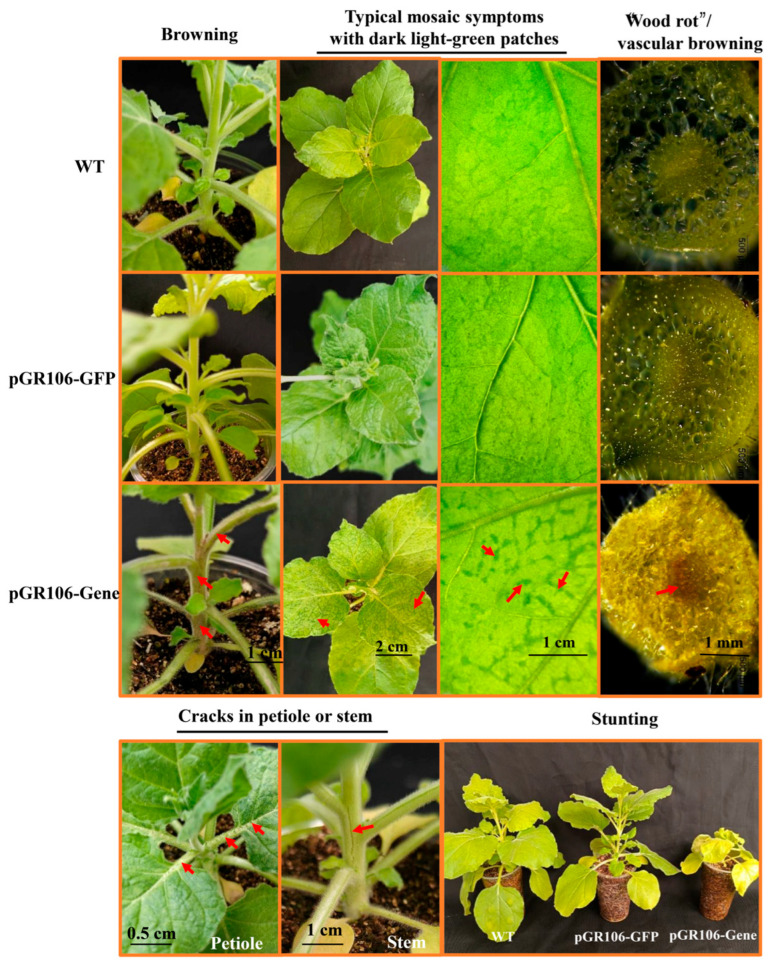
Four distinct phenotypes upon pectate lyase expression in *N. benthamiana*. Transient expression of 21 pectate lyase genes in tobacco leaves mediated by Agrobacterium tumefaciens. The genes were individually cloned into a PVX vector and expressed in tobacco. Photographs were taken 7–10 days post-infiltration. Four distinct phenotypic categories were observed. Red arrows indicate sites of visible symptom development. Symptoms with dark light-green patches or vascular browning (longitudinal section of the petiole) were examined and photographed using a stereomicroscope.

Functional characterization of *D. destructor* pectate lyase was performed through transient expression in *N. benthamiana* using a PVX-based vector system. Full-length coding sequences (including signal peptides) of 21 *Ddpel* genes of pectate lyase gene family were cloned into the pGR106 vector. Phenotypic responses were documented at 10 days post-inoculation, revealing four distinct symptom categories (Figure 5; Table S3): (Type I) Typical mosaic symptoms with dark light-green patches; (Type II) Type I symptoms superimposed with petiole maceration featuring compromised mechanical integrity—healthy petioles exhibited elastic flexion and audible fracture, while affected petioles underwent silent disintegration resembling decayed wood, accompanied by vascular browning upon sectioning and occasional dwarfing; (Type III) type I symptoms combined with longitudinal stem/petiole fissuring and severe dwarfing; and (Type IV) Type I symptoms combined with stem browning and necrosis. Based on the phenotypic hierarchy observed in *N. benthamiana*, Type I represents the basal pathology with the mildest symptoms, while Types II–IV exhibit enhanced virulence. This progression suggests that nematodes may have evolved functionally distinct pectate lyase isoforms to adapt to diverse host environments. Despite the occurrence of diverse necrotic phenotypes, no hypersensitive response was detected at the infiltration sites on *N. benthamiana* leaves.

Among the prioritized pectate lyases, DdPeL8, DdPeL10, and DdPeL14 exclusively elicited Type I symptoms in tobacco (Table 2). In contrast, DdPeL15 induced Type III pathology, DdPeL16 triggered Type II lesions, and both DdPeL18 and DdPeL19 produced Type IV necrosis (Table 2). These results indicate that alkaline DdPeLs caused more severe necrosis than acidic DdPeLs in *D. destructor*.

### 3.6. Enzymatic Activity Is a Critical Determinant of Pathogenicity

**Figure 6:**
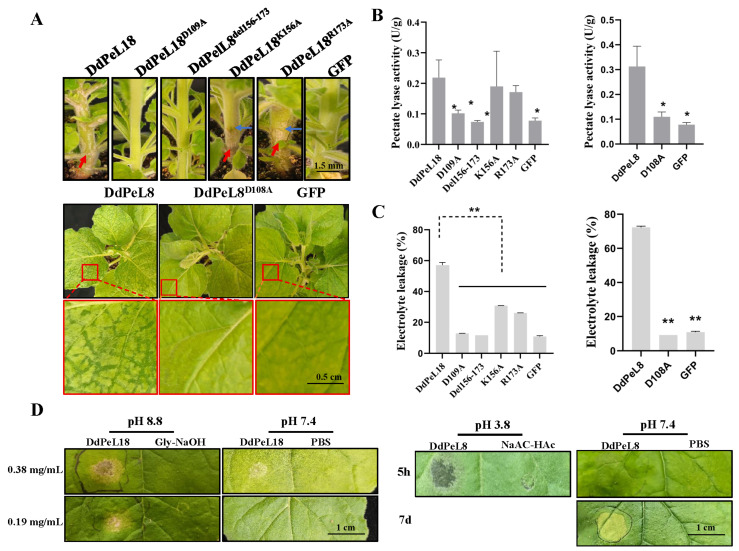
Enzymatic activity is a critical determinant of pathogenicity. (**A**) The phenotypic analysis of pectate lyase mutants in tobacco leaves via Agrobacterium-mediated transient expression. Mutations were introduced into conserved domains or key residues of acidic (DdPeL8) or alkaline (DdPeL18) pectate lyases. Red arrows indicate sites of stem browning; Blue arrows indicate the boundary between healthy and diseased regions; the red box highlights symptoms induced by the expression of an acidic pectate lyase in leaves. (**B**) The enzymatic activity of the wild-type and mutant pectate lyase proteins. (**C**) Electrolyte leakage from tobacco leaves expressing wild-type or mutant pectate lyase genes. (**D**) Symptoms on tobacco leaves following the infiltration of acidic/alkaline pectate lyase proteins dialyzed in buffers at different pH values. The yellowed and translucent areas on tobacco represent the disease lesions caused by pectate lyase. For comparisons among multiple groups, one-way analysis of variance (ANOVA) was performed, followed by Fisher’s Least Significant Difference (LSD) test for multiple comparisons. Statistical significance: *: *p* < 0.05; **: *p* < 0.01. Data are presented as mean ± standard deviation.

Our study revealed that most pectate lyases upregulated during early *D. destructor* infection were acidic isoforms, yet these induced milder necrosis in tobacco compared to alkaline isoforms. This suggested that enzymatic activity within the host microenvironment may be a key factor determining pathogenicity. To test this hypothesis, DdPeL18 (alkaline pectate lyase) and DdPeL8 (acidic pectate lyase) were selected for functional analysis. Previous reports suggest the importance of specific residues (aspartic, arginine or lysine) for PeL enzymatic activity [5,6]. Through multiple sequence alignment analyses, we identified three conserved residues (Asp109, Arg173 and Lys156; Table S5). A series of site-directed mutants targeting key catalytic residues (DdPeL18^D109A^, DdPeL18^K156A^, DdPeL18^R173A^, DdPeL18^d^^el156-173^, and DdPeL8^D108A^) were constructed. Enzymatic assays revealed that three mutants (DdPeL18^D109A^, DdPeL18^del156-173^, and DdPeL8^D108A^) exhibited significantly reduced polygalacturonic acid degradation activity compared to the wild-type proteins (Figure 6A,B). Moreover, the ability to induce cell death was completely lost in these three mutants (Figure 6A,B). For the other two mutants (DdPeL18^K156A^ and DdPeL18^R173A^), a reduced ability to induce cell death was observed (Figure 6A,B). Electrolyte leakage was used to quantify the extent of cell death [6]. Consistently, the levels of electrolyte leakage induced by these mutants were significantly lower than those induced by wild-type DdPeL18 or DdPeL8 (Figure 6C).

Given the pH dependence of enzyme activity, we further examined pathogenicity under different pH conditions. When DdPeL18 was dialyzed in its optimal pH buffer (pH 8.8) versus PBS (pH 7.4) and infiltrated into tobacco, the pH 8.8-treated protein induced significantly stronger necrosis within 48 h (Figure 6D). Similarly, DdPeL8 dialyzed at pH 3.8 caused clear tissue maceration within 5 h, while the PBS-treated control produced only faint yellowish lesions after 7 days (Figure 6D). These results highlight the importance of microenvironmental pH for enzymatic function. A dose–response assay further confirmed that the pathogenicity of DdPeL18 positively correlated with protein concentration (Figure 6D).

Further analysis of the enzymatic activities measured at pH 5.8 revealed that enzymes associated with severe necrosis (Types II–IV) generally showed higher activity (0.019–0.021 U mg^−1^) than those causing mild symptoms (Type I; 0–0.012 U mg^−1^; Table S2). Similarly, *Ddc_19181* (0.018 U mg^−1^) from *D. destructor* population B also induced more severe necrosis in tobacco than *Ddc_10541* (0.005 U mg^−1^; Table S2). Although infection has been reported to trigger apoplastic alkalinization, no significant difference in activity was observed between alkaline and acidic DdPeLs under alkaline conditions in this study.

### 3.7. pH Is Significantly Lower in Infected Sweet Potato Tissues

**Figure 7:**
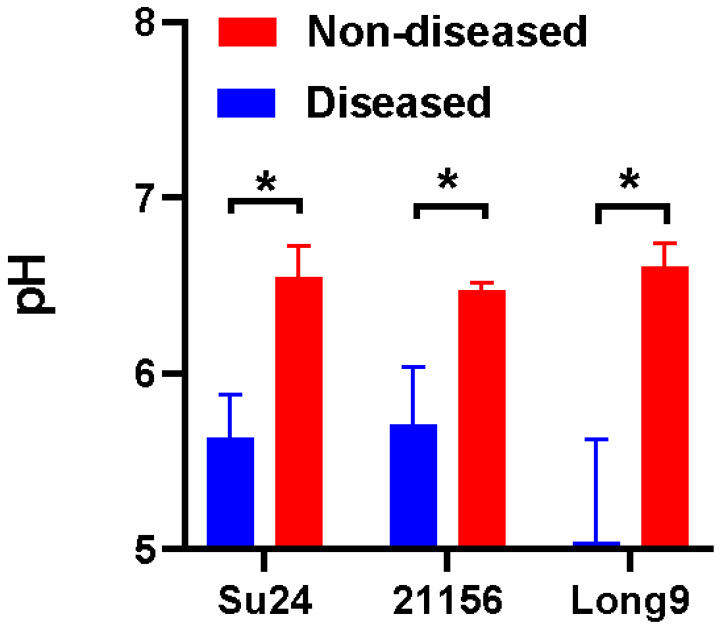
pH is significantly lower in infected sweet potato tissues (mature stage). pH measurements of healthy and *Ditylenchus destructor*-infected sweet potato tissues across three varieties: su24, 21156 (resistant), and Long9 (susceptible). Statistical significance is indicated as follows: *: *p* < 0.05.

Based on previous reports that pathogens secrete acidic substances during early infection to enhance CWDE activity [16], we examined whether *D. destructor* acidifies the host microenvironment. Direct pH measurement at early infection sites is technically difficult. Therefore, we measured pH at infection sites in severely diseased tubers at the maturity stage, considering that *D. destructor* continuously degrades cell walls without forming a fixed feeding site, and that substantial acidic secretions may occur when nematodes proliferate abundantly within tubers. Thus, the pH at the maturity stage may indirectly reflect nematode-induced acidification. We measured the pH of healthy and nematode-infected sweet potato tissues (Figure S4). Two resistant cultivars (su24 and 21156) and one susceptible cultivar (Long9) were examined. The results showed that the pH of infected tissues was significantly lower than that of healthy tissues in the susceptible cultivar (Figure 7). Moreover, the pH at the infection sites in the susceptible cultivar was significantly lower than that in the resistant cultivars (Figure 7).

## 4. Discussion

*D. destructor* is a devastating pathogen of sweet potato and ranks as the second most economically significant plant-parasitic nematode infecting potato [2,33]. Pectate lyases, critical CWDEs, play essential pathogenic roles during the early infection phase of this nematode. While prior studies have primarily focused on the functional characterization of individual *pel* genes [18,30], these enzymes exist as gene families, and the dominant PeL isoform during the initial infection time course remains unclear. Our study reveals two key phenomena: (1) The level of pectate lyase enzymatic activity plays a significant role in determining the intensity of tobacco necrosis. (2) Prior to host infection, *D. destructor* preferentially expresses *Ddpel* genes encoding pectate lyases with higher activity at pH 5.8. However, the initial infection stage triggers the upregulation of genes encoding acidic DdPeL enzymes, along with the concurrent downregulation of genes encoding alkaline DdPeL enzymes. Given the significant acidification observed in infected sweet potato tissues, we propose that *D. destructor* enhances its pathogenicity by secreting DdPeL isoforms that match the ambient pH, enabling the efficient degradation of the plant cell wall.

### 4.1. Limitations of Agrobacterium-Mediated Transient Expression in Tobacco for Screening CWDE Effector Proteins

CWDEs play a critical role during the initial stages of pathogen infection [11]. Consequently, the predominant strategy for identifying CWDEs functioning as effector proteins relies on selecting genes upregulated in transcriptomes during early infection and verifying their potential through their ability to elicit the hypersensitive response in *N. benthamiana* [7,25]. However, both this study and a previous work demonstrate that the enzymatic activity of CWDEs is a key determinant of the intensity of necrosis induction [6]. Therefore, this screening approach may primarily isolate CWDEs that exhibit high activity specifically within the *N. benthamiana* microenvironment (pH 5.8–6.2 or rising to alkaline levels upon defense-induced alkalinization). In contrast, many pathogens actively acidify the apoplast during the initial infection stage to enhance the catalytic efficiency of their acidic CWDEs. For example, *Fusarium oxysporum* secretes fusaric acid and *Sclerotinia sclerotiorum* secretes oxalic acid, which significantly boost the activity of CWDEs such as cellulases and xylanases [16]. Thus, the current strategy overlooks the dynamic regulation of CWDE activity by microenvironmental pH, particularly pathogen-induced acidification. This limitation likely leads to the systematic omission of CWDE effector proteins that function at acidic pH. This study provides clear examples: *Ddpel8* with high expression during the initial infection time course and high in vitro enzymatic activity (optimal pH 3.8) was frequently overlooked because it induced weak necrosis in *N. benthamiana* (Figure 2B). Conversely, *Ddpel18*, which strongly induces necrosis, was potentially excluded due to its downregulated expression early in the infection (Figure 2B and Table S3). Notably, the presence of four genomic copies of *Ddpel18* strongly suggests its crucial role in nematode infection. The limitations of the current CWDE effector screening strategy may therefore lead to a serious underestimation of the functional significance of such key proteins.

### 4.2. Exploration of Pectate Lyase Secretion by D. destructor

Based on the key observation from this study that enzyme activity determines the infection phenotype, and considering the significant upregulation of acidic *Ddpel* genes alongside the downregulation of alkaline *Ddpel* genes within 5 days post-inoculation, we propose a temporal regulation model for the expression and secretion of DdPeL in *D. destructor*. This model integrates the known mechanisms of environmental pH-regulated pectate lyase expression. During the pre-infection preparation stage, the nematode may constitutively express *Ddpel* genes whose optimal enzymatic pH (~5.4) matches the initial weakly acidic environment of the host apoplast (pH 5.0–6.5). This ensures a quick response when the host is encountered. In the initial phase post-inoculation, the nematode preferentially secretes DdPeL isoforms with high activity under acidic conditions while simultaneously suppressing the expression of *Ddpel* genes, such as those with optimal alkaline pH, that are not adapted to the current environmental pH. Multiple lines of evidence support this strategy: (1) The endo-polygalacturonase purified from *Alternaria alternata* (a key CWDE) exhibits optimal activity at pH 4.0–5.0 (acidic range) and remains stable at pH levels below 6.0 [14]. (2) A systematic analysis of the factors regulating the activity of fungal CWDEs reveals that a low pH (around 5.0) can enhance their binding capacity to plant cell wall polysaccharides by altering the spatial conformation of the enzymes. This pH-dependent regulatory mechanism is highly conserved among ascomycetes [34]. (3) Pathogenic fungi and bacteria, such as *B. cinerea* secreting citric acid [17], *F. oxysporum* secreting fusaric acid/inducing host proton pump activation, and *S. sclerotiorum* secreting oxalic acid, all acidify the apoplast at the infection site to activate acidic-optimum CWDEs [16,35].

When the host plant perceives cell wall degradation (e.g., triggered by acidic PeL), it rapidly activates defense responses [11]. This involves the production of reactive oxygen species (ROS) and the regulation of ion channels (such as inhibiting H^+^-ATPase or promoting anion influx) [18,19], leading to apoplast alkalinization. This alkaline shift inactivates acidic-optimum PeL enzymes. Therefore, although tobacco tissue is slightly acidic under normal conditions, the localized alkalinization of the apoplast during defense responses may create an environment more favorable for the activity of alkaline PeLs, thereby accounting for their induction of more severe necrosis. As an adaptive response, the nematode switches to secreting and expressing PeL isoforms with optimal alkaline pH during the mid to late stages of infection. Their functions may include the following: (1) Sustained degradation: alkaline PeL is activated to maintain the cell wall breakdown process when plant tissue necrosis or significant defensive alkalinization elevates the local pH [11,35,36]. (2) Substrate-specific degradation: alkaline PeL may preferentially acts on low-methyl-esterified polygalacturonic acid. As infection progresses, the degree of pectin de-methylation mediated by early-acting enzymes (e.g., pectin methylesterase) increases, providing suitable substrates for alkaline PeL and thereby facilitating deep pectin degradation [37].

The probable reasons for the overexpression of acidic DdPeL during the initial phase are speculated to be as follows: Firstly, highly methyl-esterified homogalacturonan tends to form a degradation-resistant gel network under neutral to alkaline conditions, whereas an acidic environment promotes its solubilization. Secondly, acidic DdPeL may target the highly methyl-esterified pectin, which is abundant during early infection, enabling initial penetration. In contrast, alkaline DdPeL may adapt to the low-methyl-esterified substrates that accumulate in the middle and late stages. Thirdly, regarding environmental compatibility, the initial weakly acidic host apoplast (pH 5.0–6.5) naturally aligns with the optimal activity requirements of acidic DdPeL.

### 4.3. Limitations and Future Perspectives

While this study provides initial insights into the expression dynamics of acidic/alkaline pectate lyases during early *D. destructor* infection, several limitations warrant consideration. First, our analysis was restricted to only seven pectate lyases, omitting a systematic functional assessment of other CWDEs, thus limiting the comprehensiveness of our conclusions. Second, the current apoplastic pH measurements were obtained from sweet potato tissues exhibiting visible disease symptoms, which may not accurately reflect microenvironmental conditions during the initial infection stages. Future investigations should employ in vivo pH imaging to spatiotemporally monitor apoplastic pH fluctuations throughout nematode infection while concurrently tracking acidic/alkaline DdPeL expression patterns. Furthermore, we cannot rule out the potential confounding effects from nematode-associated fungi contributing to observed pH reductions. To address these gaps, future work should: (1) delineate the spatiotemporal expression profiles of acidic/alkaline DdPeLs using combinatorial pH imaging and immuno-localization techniques and (2) determine whether this pH-dependent regulatory mechanism extends to other migratory nematode taxa.

## 5. Conclusions

This study reveals that *D. destructor* preferentially expresses genes encoding pectate lyases with higher enzymatic activity prior to host infection. In the initial phase post-inoculation, the nematode shifts its expression profile, upregulating genes encoding acidic DdPeLs while downregulating alkaline isoforms. Our findings further demonstrate that the enzymatic activity is essential for pathogenicity. Previous studies have reported that phytopathogens can secrete organic acids during the initial infection phase, leading to localized acidification of the host microenvironment. Given the significant decrease in pH observed in infected sweet potato tissues (in the maturity stage), we propose that the nematode may actively secrete acidic substances during early infection to acidify the host microenvironment. This acidification likely enhances the activity of acidic DdPeLs, thereby promoting plant cell wall degradation.

## Figures and Tables

**Table 1:** Optimal pH for DdPeL activity and its effects on *Nicotiana benthamian*.

Protein	Optimal pH for Enzyme Activity	Grouping by Optimum pH	Phenotype of *N. benthamian*
DdPeL18	9	Alkalinity	Browning in stem
DdPeL15	9	Alkalinity	Cracks in petiole or stem
DdPeL19	8.8	Alkalinity	Browning in stem
DdPeL16	9	Alkalinity	“Brittle as Rotten Wood”
DdPeL14	7.4	Neutrality	Typical mosaic symptoms with dark light-green patches
DdPeL8	3.8	Acidity	Typical mosaic symptoms with dark light-green patches
DdPeL10	5.8	Acidity	Typical mosaic symptoms with dark light-green patches

**Table 2:** Phenotypic changes in *Nicotiana benthamiana* plants infected with recombinant potato virus X carrying *Ddpel* genes.

Gene	Phenotypes in Tobacco					Main Phenotype
	Typical Mosaic Symptoms with Dark Light-Green Patches	Browning in Stem	Cracks in Petioles or Stems	“Brittle as Rotten Wood”	Stunting	
*Ddpel8*	√	×	×	×	×	Typical mosaic symptoms with dark light-green patches (Type I)
*Ddpel14*	√	×	×	×	×	Typical mosaic symptoms with dark light-green patches (Type I)
*Ddpel10*	√	×	×	×	×	Typical mosaic symptoms with dark light-green patches (Type I)
*Ddpel15*	√	×	√	√	√	Cracks in petiole or stem (Type III)
*Ddpel16*	√	×	×	√	√	“Brittle as Rotten Wood” (Type II)
*Ddpel18*	√	√	×	×	√	Browning in stem (Type IV)
*Ddpel19*	√	√	×	×	√	Browning in stem (Type IV)

## Data Availability

The data presented in this study are available on request from the corresponding authors due to privac.

## Supplementary Materials

The following supporting information can be downloaded at: https://www.mdpi.com/article/10.3390/microorganisms14040829/s1, Figure S1: Prokaryotic expression and purification of pectate lyase; Figure S2: Correlation analysis between the basal expression levels of pectate lyase genes in the absence of *Ditylenchus destructor* infection and the corresponding enzymatic activities measured at different pH values; Figure S3: Measurement of pH in potato, tobacco, carrot, and sweet potato tissues; Figure S4: Healthy and diseased sweet potato. Table S1: Physicochemical properties of DdPeLs; Table S2: Enzyme activity of DdPeL at different pH values; Table S3: Phenotypic changes in *Nicotiana benthamiana* plants infected with recombinant potato virus X carrying *Ddpel* genes; Table S4: Primers in this study; Table S5: Sequence alignment analyses.
