# Supplementary material for: Divergent Expression of Acidic and Alkaline Pectate Lyases in Ditylenchus destructor During Initial Infection Time Course
Source: Microorganisms. 2026 Apr 4;14(4):829. doi: 10.3390/microorganisms14040829 (PMC13119071; doi:10.3390/microorganisms14040829)
Supplement: Supplementary file 1 [file microorganisms-14-00829-s001.zip › Figure s1-s4.pdf]

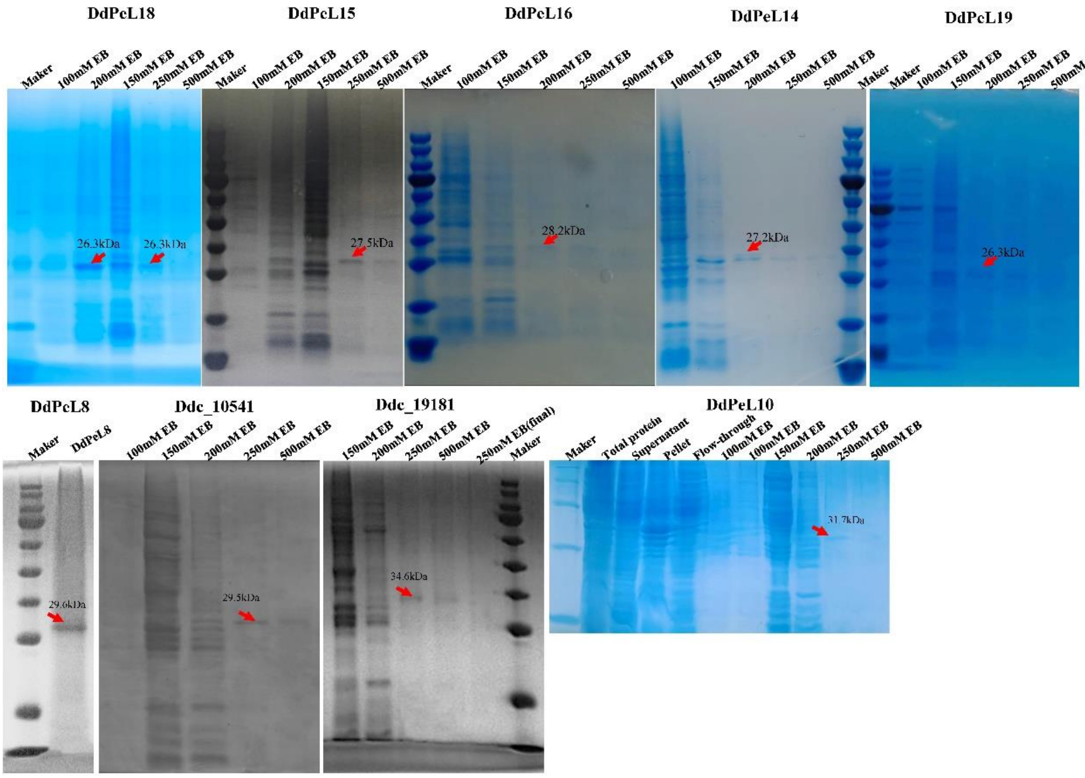

Fig. S1 Prokaryotic expression and purification of pectate lyase

Analysis of recombinant pectate lyase expression and purification. The red arrow indicates the molecular weight of the target protein, as confirmed by SDS-PAGE. The elution profile shows the purification of the protein using an imidazole gradient ranging from 100 mM to 500 mM.

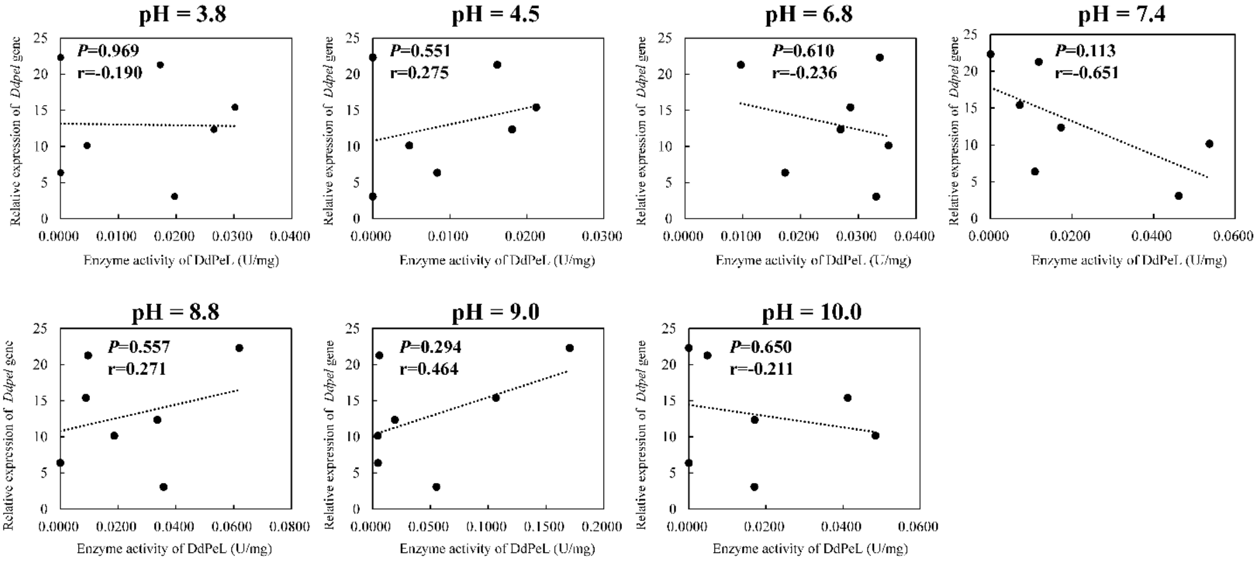

Fig. S2 Correlation analysis between the basal expression levels of pectate lyase genes in the absence of *Ditylenchus destructor* infection and the corresponding enzymatic activities measured at different pH.

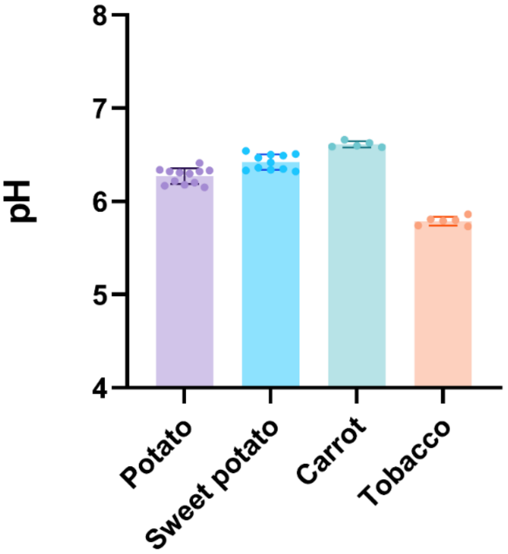

Fig S3. Measurement of pH in potato, tobacco, carrot, and sweet potato tissues

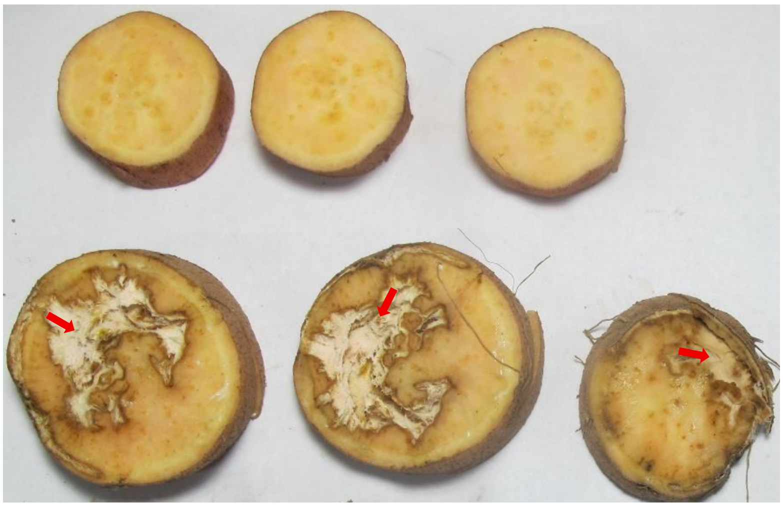

Fig S4. Healthy and diseased sweet potato.

The red arrow indicates the diseased part
